# Supplementary material for: Marine heatwaves disrupt ecosystem structure and function via altered food webs and energy flux
Source: Nat Commun. 2024 Mar 13;15:1988. doi: 10.1038/s41467-024-46263-2 (PMC10937662; doi:10.1038/s41467-024-46263-2)
Supplement: Supplementary file 3 — Description of Additional Supplementary Files [file 41467_2024_46263_MOESM3_ESM.pdf]

Description of Additional Supplementary Files

File Name: Supplementary Data 1

Description: **Ecosystem model parameter comparison.** Ecopath parameterization of pre-MHW and MHW models. Years represent focus dates for representing biomass in the system (see Methods). TL = estimated trophic level, Biomass = calculated (or estimated) average biomass density in ecosystem (mt/km2), EE = ecotrophic efficiency, PB = biomass-specific production rate, CB = biomass-specific consumption rate. EE values are estimated by the Ecopath master equation (see Gomes et al. 2022; 30), except those in which biomass was estimated (EE in bold are fixed). See data and code for .csv version.
