# Supplementary material for: Marine heatwaves disrupt ecosystem structure and function via altered food webs and energy flux
Source: Nat Commun. 2024 Mar 13;15:1988. doi: 10.1038/s41467-024-46263-2 (PMC10937662; doi:10.1038/s41467-024-46263-2)
Supplement: Supplementary file 5 — Reporting Summary [file 41467_2024_46263_MOESM5_ESM.pdf]

nature portfolio

Corresponding author(s): Dylan G. E. Gomes

Last updated by author(s): 1/31/2024

Reporting Summary

Nature Portfolio wishes to improve the reproducibility of the work that we publish. This form provides structure for consistency and transparency in reporting. For further information on Nature Portfolio policies, see our Editorial Policies and the Editorial Policy Checklist.

Statistics

For all statistical analyses, confirm that the following items are present in the figure legend, table legend, main text, or Methods section.

n/a	Confirmed	
☐	☒	The exact sample size (*n*) for each experimental group/condition, given as a discrete number and unit of measurement
☐	☒	A statement on whether measurements were taken from distinct samples or whether the same sample was measured repeatedly
☐	☒	The statistical test(s) used AND whether they are one- or two-sided<br>*Only common tests should be described solely by name; describe more complex techniques in the Methods section.*
☒	☐	A description of all covariates tested
☐	☒	A description of any assumptions or corrections, such as tests of normality and adjustment for multiple comparisons
☐	☒	A full description of the statistical parameters including central tendency (e.g. means) or other basic estimates (e.g. regression coefficient) AND variation (e.g. standard deviation) or associated estimates of uncertainty (e.g. confidence intervals)
☐	☒	For null hypothesis testing, the test statistic (e.g. *F*, *t*, *r*) with confidence intervals, effect sizes, degrees of freedom and *P* value noted<br>*Give P values as exact values whenever suitable.*
☒	☐	For Bayesian analysis, information on the choice of priors and Markov chain Monte Carlo settings
☒	☐	For hierarchical and complex designs, identification of the appropriate level for tests and full reporting of outcomes
☒	☐	Estimates of effect sizes (e.g. Cohen's *d*, Pearson's *r*), indicating how they were calculated

Our web collection on statistics for biologists contains articles on many of the points above.

Software and code

Policy information about availability of computer code

Data collection	We used custom code in MATLAB (R2021a) to build the models and run simulations, available at https://doi.org/10.5281/zenodo.8121889
Data analysis	We used custom code in R (version 4.2.2) to organize, visualize, and analyze ecosystem models and simulation outputs, available at https://doi.org/10.5281/zenodo.8121889

For manuscripts utilizing custom algorithms or software that are central to the research but not yet described in published literature, software must be made available to editors and reviewers. We strongly encourage code deposition in a community repository (e.g. GitHub). See the Nature Portfolio guidelines for submitting code & software for further information.

Data

Policy information about availability of data

All manuscripts must include a data availability statement. This statement should provide the following information, where applicable:

- Accession codes, unique identifiers, or web links for publicly available datasets
- A description of any restrictions on data availability
- For clinical datasets or third party data, please ensure that the statement adheres to our policy

All data, code, and materials used in the analysis are available in a long-term data repository at: https://doi.org/10.5281/zenodo.8121889

Research involving human participants, their data, or biological material

Policy information about studies with human participants or human data. See also policy information about sex, gender (identity/presentation), and sexual orientation and race, ethnicity and racism.

Reporting on sex and gender	N/A
Reporting on race, ethnicity, or other socially relevant groupings	N/A
Population characteristics	N/A
Recruitment	N/A
Ethics oversight	N/A

Note that full information on the approval of the study protocol must also be provided in the manuscript.

Field-specific reporting

Please select the one below that is the best fit for your research. If you are not sure, read the appropriate sections before making your selection.

- [ ] Life sciences
- [ ] Behavioural & social sciences
- [x] Ecological, evolutionary & environmental sciences

For a reference copy of the document with all sections, see nature.com/documents/nr-reporting-summary-flat.pdf

Ecological, evolutionary & environmental sciences study design

All studies must disclose on these points even when the disclosure is negative.

Study description	We built two end-to-end ecosystem models of the Northern California Current marine ecosystem. One model is parameterized with data that were collected prior to recent marine heatwaves (i.e., prior to 2014), and the other model was updated with data collected during and after recent marine heatwaves (i.e., from 2014 onwards). While the models themselves are not statistical in design, we visually and statistically compare ecosystem models, changes in energy flows, and the importance of various functional groups as consumers and producers within the system. In order to statistically compare models, we rely on re-sampling uncertainty in model parameters as Monte Carlo models (see Sampling strategy), thus there are no experimental units or replicates as one might have in a experimental or controlled study.
Research sample	The research sample for this study is the entire ecosystem within the Northern California Current taken from two time periods, meant to represent the actual marine ecosystem within the Northern California Current before and after the onset of the 2014 marine heatwaves. We chose this as the sample because we are interested in differences in the ecosystem between two states. Two different models were parameterized with many years of data on 86 functional groups. Uncertainties around these parameterizations were also assigned, such that we can randomly sample about all ecosystem parameter values with Monte Carlo models (see sampling strategy).
Sampling strategy	For statistical tests in the model, 100 Monte Carlo (MC) models were built for each of the two ecosystem models in this study. Each Monte Carlo model is a different realization of the initial ecosystem model that was parameterized, which was realized through random sampling (via a normal distribution) around all ecosystem model parameters. We chose 100 MC models because it is a large enough number to ensure that uncertainty is incorporated throughout each component of the ecosystem model, yet not so large as to lead to spurious 'statistically significant' results, since the likelihood of obtaining significant results increases with larger sample sizes, even with meaningless effect sizes.
Data collection	Data were initially collected by various long-term field surveys (conducted by NOAA Fisheries and Oregon State University) or for other studies (sources of literature). For this particular study, gathering the data consisted of contacting authors of previous work, contacting database managers, and searching publicly available information for sources of data for inclusion into the ecosystem model. The first two authors (Dylan G.E. Gomes and James J. Ruzicka) gathered all data (from existing sources) for inclusion into the ecosystem model.
Timing and spatial scale	For the pre-MHW ecosystem model, we incorporated available data from prior to 2012. Many of the long-term datasets began in the late 1990s or early 2000s, but a few sources of literature date back to the late 1960s and early 1970s. For the post-MHW ecosystem model, we updated the model with available data from 2014 - 2022. For cases where new information was not available, pre-MHW values were used for the post-MHW model. For all data incorporation, we focused on data that were collected from within the Northern California Current (from 40.8 degrees N to 48.34 degrees N and from 0 m depth to 1280 m isobath depth; offshore of Washington, Oregon, and Northern California in the United States), but some information (e.g., physiological parameters for some functional groups) was taken from nearby marine ecosystems. Detailed description of the data used for these models are found at the following links: https://doi.org/10.1016/j.pocean.2012.02.002, https://doi.org/10.1101/2022.12.28.522165, and https://doi.org/10.5281/zenodo.7079777
Data exclusions	No data were excluded from this analysis, as no new field data were collected.

Reproducibility	All data and code used to generate the model and reproduce all analyses and visualizations are found here: https://doi.org/10.5281/zenodo.8121889 and here: https://doi.org/10.5281/zenodo.7079777. The model and analysis code have been tested on multiple machines, and are found to reproduce the same results as reported here.
Randomization	Our experiments were simulated, where randomization was included during the Monte Carlo model generation process. Each model parameter was selected for Monte Carlo models by drawing from a normal distribution with a mean and standard deviation defined by the raw data and general ecosystem modelling assumptions of uncertainty.
Blinding	Data were incorporated into ecosystem models by years, without any exclusion criteria for data. Additionally, we did not have formal hypotheses or predictions for what the differences between ecosystem models would be. In this case blinding is not useful. Instead, we explore differences in the entire ecosystem, energy flows, and functional groups' contributions to and consumption of upper and lower trophic levels, respectively, between pre- and post-marine heatwave periods of time.

Did the study involve field work? ☐ Yes ☒ No

Reporting for specific materials, systems and methods

We require information from authors about some types of materials, experimental systems and methods used in many studies. Here, indicate whether each material, system or method listed is relevant to your study. If you are not sure if a list item applies to your research, read the appropriate section before selecting a response.

Materials & experimental systems

n/a	Involved in the study
☒	☐ Antibodies
☒	☐ Eukaryotic cell lines
☒	☐ Palaeontology and archaeology
☒	☐ Animals and other organisms
☒	☐ Clinical data
☒	☐ Dual use research of concern
☒	☐ Plants

Methods

n/a	Involved in the study
☒	☐ ChIP-seq
☒	☐ Flow cytometry
☒	☐ MRI-based neuroimaging
